# Effects of Caffeine on Dental Mesenchymal Stem Cells: Implications for Regenerative Applications

**DOI:** 10.3390/jfb16090314

**Published:** 2025-08-29

**Authors:** Axel Alejandro Lugo-Sanchez, Patricia Alejandra Chavez-Granados, Carlos A. Jurado, Ziyad Allahem, Jorge Emmanuel Ramirez-Lopez, Christian Andrea Lopez-Ayuso, Benjamin Aranda-Herrera, Abdulrahman Alshabib, Rene Garcia-Contreras

**Affiliations:** 1 Interdisciplinary Research Laboratory, Nanostructures and Biomaterials Area, National School of Higher Studies (ENES) Leon Unit, National Autonomous University of Mexico (UNAM), Leon 37684, Mexico; 2 Division of Operative Dentistry, Department of General Dentistry, College of Dentistry, The University of Tennessee Health Science Center, Memphis, TN 38103, USA; 3 School of Dental Medicine, Ponce Health Sciences University, Ponce, PR 00732, USA; 4 Department of Restorative Dental Science, College of Dentistry, King Saud University, Riyadh 11545, Saudi Arabia

**Keywords:** caffeine, DPSC, metabolic activity, proliferation, characterization, inflammation, bacteria

## Abstract

Periodontal therapy remains a complex task in dentistry as current methodologies often tend to induce tissue repair rather than regeneration. Caffeine is an alkaloid found in multiple natural sources, which has been reported to have multiple beneficial effects, such as promoting adipogenic differentiation, a key factor in tissue regeneration. Unfortunately, it has also been reported to decrease cell viability and reduce osteogenic and chondrogenic differentiation, both of which play an important role in regenerative medicine. In this study, we aimed to find a non-cytotoxic dose of purified caffeine over dental pulp stem cells (DPSCs) that could provide its beneficial effects over adipogenesis, while reducing the negative effect upon osteogenesis and chondrogenesis. Additional experiments were conducted to determine its impact upon the expression of pro-inflammatory enzymes, and antibacterial assays to assess a potential antibacterial effect. The results attested that purified caffeine at a dose of 8.03 μM holds no viability reduction effect, nor has any impact on the expression of pro-inflammatory enzymes, promotes adipogenic differentiation, and does not negatively affect osteogenic or chondrogenic differentiation, with any antibacterial effect against *Streptococcus mutans*, *Escherichia coli*, and *Staphylococcus aureus*. These findings suggest that purified caffeine at a dose of 8.03 μM has the potential to aid in the field of regenerative dentistry.

## 1. Introduction

Periodontitis is a chronic infectious disease that compromises the supporting structures of the teeth. If left untreated, it leads to progressive destruction of periodontal tissues and may ultimately result in tooth loss due to lack of structural support [1,2]. It is currently one of the major causes of tooth loss worldwide [2]. Periodontal therapy aims to restore the tissue affected by periodontal disease; this includes the lost gingival and bone tissue, as well as the re-establishment of the periodontal fibers on the root surfaces.

The disease typically begins as gingivitis, a reversible condition that can be managed with proper oral hygiene. However, if not adequately managed, it can progress to periodontitis. Originally classified at the 1999 International Workshop on Periodontal Diseases, periodontitis was redefined in 2017 to incorporate advancements in scientific understanding and classification criteria [3]. Its global prevalence is estimated to affect approximately 50% of the population, making it a significant public health concern.

Conventional periodontal therapy focuses on halting disease progression and repairing damaged tissues. This includes the restoration of lost gingival and bone tissue, and the re-establishment of periodontal fibers on the root surfaces. Despite over four decades of clinical research and experience, most current treatments prioritize repair rather than true biological regeneration [4].

This therapeutic limitation has stimulated the development of novel regenerative approaches. Emerging strategies involve the use of stem cells (SCs), nanomaterials, genetic engineering, and bioactive natural compounds, including herbal extracts, to promote tissue regeneration and restore periodontal function more effectively [3].

Effective tissue regeneration requires specific cells to promote the differentiation and growth of new tissue. Additionally, the control of microbial fauna is essential to mitigate infection and facilitate periodontal therapy. Ideal periodontal regeneration has been described as an idealistic goal, as it requires the successful integration of multiple complex biological factors [1,3,4,5].

Caffeine (C_8_H_10_N_4_O_2_; 1, 3, 7-trimethylxanthine) [6] is a methylxanthine alkaloid naturally found in various plants, leaves, fruits, and seeds, and is widely consumed globally. Several studies have reported its beneficial biological effects, including antioxidant capacities and inhibition of COX-2, an enzyme involved in the inflammatory process [7,8]. It has also been reported to possess antibacterial activity [9,10,11]. Nonetheless, its influence on adipogenic differentiation in stem cells (SCs) remains controversial, possibly due to the specific cell lines employed in different studies [12,13,14]. Furthermore, some undesirable effects have been documented, such as suppression of cell viability and osteogenic differentiation [8,12,15]. Antagonistic effects have been documented regarding its effects upon the growth of cartilage, with studies reporting promotion of genes related to chondrogenic differentiation, at the expense of increased apoptosis [15].

SCs are defined by their high clonogenic potential, self-renewal capabilities, and potential to differentiate into multiple cell types. Their primary function is to regenerate and replace senescent or damaged cells [16]. Among these, dental pulp stem cells (DPSCs) are mesenchymal stem cells derived from the neurovascular bundle of permanent teeth. First identified by Gronthos in 2000 [17], DPSCs have since garnered significant interest due to their accessibility and minimally invasive collection procedures, which result in low morbidity [18]. These multipotent stem cells can differentiate into multiple lineages, including adipogenic, chondrogenic, and osteogenic cells [19,20]. They also play a crucial role in the production of odontoblasts [21]. DPSCs exhibit superior cell proliferation, along with higher telomerase activity, compared to bone marrow mesenchymal stem cells (BMMSCs), the most extensively studied SCs. They are capable of generating colony-forming units–fibroblastic (CFU-Fs), with reports suggesting that their CFU-Fs capacity is five times greater than that of BMMSCs [20]. These characteristics, along with other reported biological advantages, make DPSCs a promising tool for treating various diseases, including periodontal disease and alveolar bone atrophy [16,18,19].

In this study, the objective was to investigate the effects of caffeine on dental pulp stem cells (DPSCs). The study specifically evaluated the compound’s effect on cell metabolic activity and proliferation using the MTT assay, aiming to identify a concentration that does not adversely affect cell viability or growth. The assay reflects reductions in metabolic activity, which serve as an indirect indicator of impaired cell proliferation. The potential antibacterial properties of caffeine were examined by conducting bacterial growth analyses against *Streptococcus mutans*, *Escherichia coli*, and *Staphylococcus aureus*. Additionally, cell differentiation into adipogenic, chondrogenic, and osteogenic lineages was induced, with the aim of replicating previously reported promotion of adipogenesis and chondrogenesis while avoiding the inhibitory effects on osteogenic pathways. Finally, a Western blot assay was performed to determine caffeine’s impact on the expression of the inflammatory-related enzymes COX-1 and -2.

## 2. Materials and Methods

### 2.1. DPSCs Culture

The isolation, culture, and characterization of DPSCs were performed in accordance with the principles of the Declaration of Helsinki and the protocols approved by the Bioethics Committee of ENES Leon (authorization code CE_16 004_SN), as previously reported [22]. Cultures were provided by the LII, ENES León cell bank, originally derived from healthy third molars (obtained from 16-year-old patients) indicated for odontectomy, which were free of pulpal and periapical pathology. Pulp tissue was aseptically extracted, sectioned into 1 mm × 1 mm explants, and cultured in MEM (Sigma-Aldrich, St. Louis, MO, USA) supplemented with 20% FBS (Gibco, Thermo Fisher Scientific, Waltham, MA, USA), 1% glutamine (Gibco, Thermo Fisher Scientific, Waltham, MA, USA), and 1% antibiotic (Sigma-Aldrich, St. Louis, MO, USA) at 37 °C, 5% CO_2_, and 95% humidity for 21 days.

### 2.2. Reduction in Cell Metabolic Assay

The DPSCs were subcultured in 96-well plates, washed with PBS, and detached using a 0.25% trypsin-0.025% EDTA-2Na solution. Cell inoculation was assessed using the trypan blue exclusion method with a hemocytometer under phase-contrast microscopy, adjusting the seeding density to 1 × 10^6^ cells/well. A stock solution of 16.06 μM purified caffeine was prepared and used to perform seven serial 1:1 dilutions, resulting in final concentrations ranging from 0 to 8.03 μM. Cells were incubated with these caffeine dilutions in fresh medium for 24 h under standard culture conditions.

Cell viability was evaluated via MTT assay. Cells were incubated with 0.2 mg/mL MTT in MEM for 4 h, after which the resulting formazan crystals were solubilized using DMSO for 30 min. Absorbance was measured at 570 nm using a microplate spectrophotometer (Multiskan GO, Thermo Fisher Scientific, Waltham, MA, USA). Cell viability classification was conducted in accordance with ISO 10993-5 guidelines [23].

### 2.3. DPSCs Metabolic Activity

Cell proliferation was evaluated using the MTT [3-(4,5-dimethylthiazol-2-yl)-2,5-diphenyltetrazolium bromide] assay following previously established protocols. Briefly, DPSCs were seeded in 96-well plates at a density of 1 × 10^6^ cells/well and allowed to adhere overnight under standard culture conditions (37 °C, 5% CO_2_, 95% humidity). The cells were then treated with purified caffeine at final concentrations of 4.01 μM and 8.03 μM, while untreated cultures served as the control group. Treatments were maintained for 3, 7, 14, and 21 days, with medium changes performed every 48 h to ensure nutrient replenishment and compound stability.

At each time point, 20 μL of MTT solution (5 mg/mL in PBS) was added to each well and incubated for 4 h to allow mitochondrial dehydrogenases in viable cells to reduce the MTT to insoluble formazan crystals. The supernatant was then carefully removed, and the formazan was solubilized with 150 μL of dimethyl sulfoxide (DMSO) per well. Absorbance was measured at 570 nm using a microplate reader (Multiskan GO, Thermo Fisher Scientific, Waltham, MA, USA). Proliferation data were normalized to the mean absorbance of the control group for each corresponding time point to enable comparison across all days.

### 2.4. Antibacterial Activity

The antimicrobial activity of purified caffeine was evaluated using the Mueller–Hinton broth microdilution technique. Three bacterial strains were employed: *Streptococcus mutans*, *Escherichia coli*, and *Staphylococcus aureus*.

Mueller–Hinton broth was prepared following the manufacturer’s specifications. Six well-isolated colonies from a 24 h culture of each bacterial strain were selected and transferred to 15 mL of Mueller–Hinton broth. The cultures were incubated at 37 °C with agitation for 24 h to obtain an active bacterial suspension. The resulting suspension was adjusted to a 0.5 McFarland turbidity standard (~1.5 × 10^8^ CFU/mL), and a 30 μL aliquot was subsequently diluted 1:1000 in fresh Mueller–Hinton broth to obtain the working bacterial inoculum. Purified caffeine was then added at various concentrations ranging from 0 to 8.03 μM. The mixtures were incubated for 4 h at 37 °C under static conditions.

To assess viability following treatment, an MTT assay was employed, dissolved in PBS. The metabolic activity of surviving bacteria, indicated by the reduction of MTT to formazan, was determined by measuring absorbance at 570 nm with a microplate spectrophotometer.

### 2.5. Differentiation of DPSCs into Adipogenic, Chondrogenic, and Osteogenic Lineages

Cells were seeded into a 24-well plate at a concentration of 1 × 10^6^ cells/well in MEM with 8.03 μM of purified caffeine for 3 days. Differentiation media for adipogenic, chondrogenic, and osteogenic differentiation were added. The differentiation culture medium was changed every two days. 

Differentiation was assessed qualitatively by evaluating the presence of lineage-specific markers under phase-contrast microscopy. The detailed composition and incubation times for each differentiation protocol are summarized in Table 1.

### 2.6. Western Blotting

Protein concentration was determined using the Bradford method. For the inflammatory stimulus, IL-33 was added to the cell cultures, which were then incubated for 3 h under standard culture conditions (37 °C, 5% CO_2_, 95% humidity). Following stimulation, cells were treated with purified caffeine at a final concentration of 8.03 μM.

For protein extraction, cultures were lysed in the presence of a protease inhibitor cocktail tablet (Roche Diagnostics, Indianapolis, IN, USA), subjected to sonication for 10 s, and centrifuged at 12,000 rpm for 10 min at 4 °C. Protein concentration in the lysates was quantified, and equal amounts of protein from each sample were separated by 8% SDS–polyacrylamide gel electrophoresis (SDS-PAGE) and transferred to polyvinylidene difluoride membranes (PVDF; Immobilon®-P Transfer Membranes, Sigma-Aldrich). Membranes were blocked with 3% skim milk under stirring conditions for 2 h at 25 °C and subsequently incubated overnight at 4 °C with primary monoclonal antibodies against COX-1 or COX-2 (Santa Cruz Biotechnology, Dallas, TX, USA) or β-actin (Sigma-Aldrich), each diluted 1:1000 in 3% blocking solution.

After washing, membranes were incubated for 1 h at room temperature with horseradish peroxidase (HRP)-conjugated polyclonal anti-mouse IgG secondary antibodies (Sigma-Aldrich) diluted 1:1000 in 3% blocking solution. Protein–antibody complexes were visualized by chemiluminescence using Clarity Max™ Western ECL substrate (Bio-Rad, Hercules, CA, USA), imaged with a C-Digit® Blot scanner (Li-Cor, Lincoln, NE, USA), and analyzed using Image Studio Version 4.0 software. Densitometric quantification of protein bands was performed using ImageJ software version 1.54p (LOCI University of Wisconsin, Madison, WI, USA), with intensities normalized to the corresponding β-actin signal from untreated controls.

### 2.7. Statistical Analysis

Statistical analyses were performed using GraphPad Prism version 10 (GraphPad Software, San Diego, CA, USA). Data were analyzed by one-way analysis of variance (ANOVA) followed by Tukey’s post hoc test to determine differences between groups. Densitometric quantification of Western blot bands was conducted using ImageJ software version 1.54p (LOCI University of Wisconsin, Madison, WI, USA) (NIH, Bethesda, MD, USA). Results are expressed as mean ± standard error of the mean (SEM), and a *p*-value < 0.05 was considered statistically significant.

## 3. Results

### 3.1. Reduction in Cell Metabolic Assay

To determine the viable doses of purified caffeine on DPSCs, metabolic activity experiments were conducted. An MTT assay with UV–VIS spectrophotometry was conducted on DPSCs treated with eight concentrations of purified caffeine: 0–8.03 μM. 

**Figure 1:**
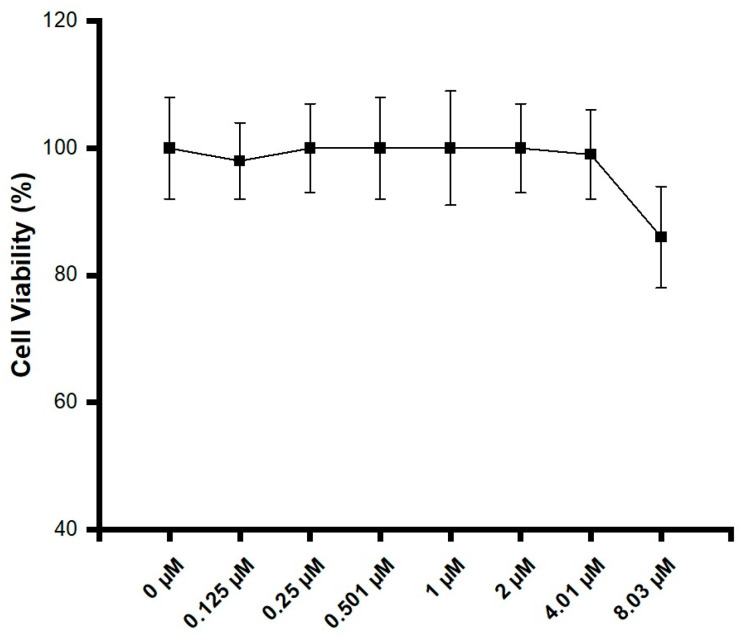
Metabolic activity of DPSCs exposed to caffeine. Cells were treated with purified caffeine doses using a stock concentration of 16.06 μM, and seven successive 1:1 dilutions were performed, ranging from 0 μM to 8.03 μM. Cell viability was evaluated by MTT assay after 24 h of caffeine exposure. Data are expressed as the percentage of control ± S.D. Statistical significance was determined using one-way ANOVA. No statistically significant differences between groups were observed.

As illustrated in Figure 1, no significant reduction in cell viability was observed across the tested concentrations. However, at a concentration of 8.03 μM, purified caffeine started to show the first signs of a decrease in cell viability. Nonetheless, at an 86% cell viability range, 8.03 μM remains inside the “non-cytotoxic” category established by ISO 10993-5 [23]. As such, 8.03 μM and 4.01 μM of purified caffeine were selected to be used in proliferation experiments.

### 3.2. DPSCs Metabolic Activity

**Figure 2:**
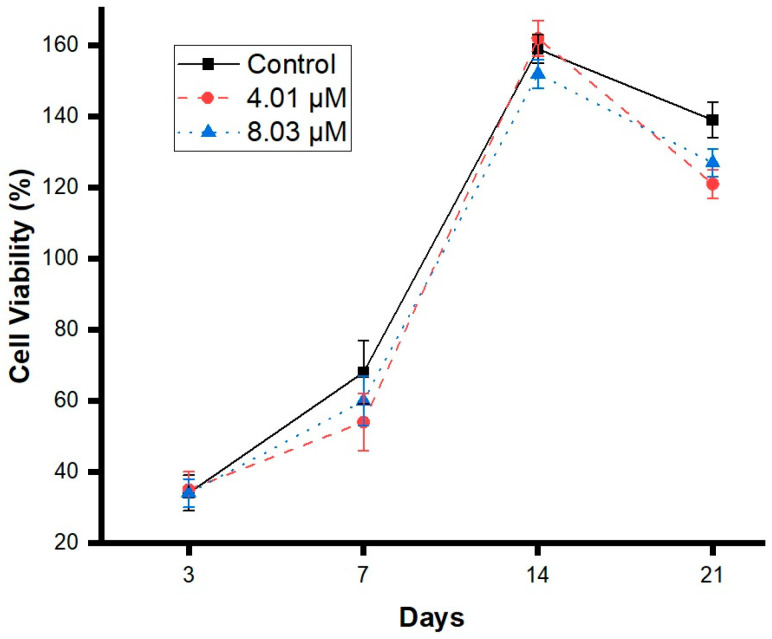
Metabolic activity of DPSCs exposed to caffeine. Cells were treated with purified caffeine at doses of 4.01 μM and 8.03 μM. Cell viability was evaluated by MTT assay after 3, 7, 14, and 21 days. (Control = 0 μM). Data are expressed as the percentage of the mean control ± S.D. Statistical significance was determined using one-way ANOVA. No statistically significant differences between groups were observed.

Based on the results of the metabolic activity assay, the two highest concentrations of purified caffeine were selected for further experiments: 8.03 μM and 4.01 μM, respectively. Cell proliferation was assessed via MTT assay under UV–VIS spectrophotometry at 3, 7, 14, and 21 days. As shown in Figure 2, the tested caffeine doses did not result in significant decreases in cell proliferation compared to the control group.

### 3.3. Antibacterial Effect 

**Figure 3:**
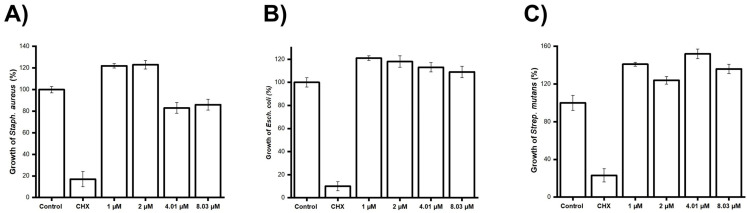
Mueller–Hinton broth microdilution assay against *Staphylococcus aureus*, *Escherichia coli*, and *Streptococcus mutans* exposed to caffeine. Bacteria were standardized using the McFarland standard. The bacteria were exposed to doses of purified caffeine ranging from 0 to 8.03 μM. Cell viability was evaluated via MTT assay. (**A**) *Staphylococcus aureus*, (**B**) *Escherichia coli*, (**C**) *Streptococcus mutans*. (Control − = bacteria; Control + = Chlorhexidine). Data are expressed as the percentage of the negative control ± S.D. Statistical significance was determined using one-way ANOVA. No antibacterial effect was observed.

A Mueller–Hinton broth microdilution assay was performed to evaluate the effect of purified caffeine at doses of 1–8.03 μM against *Staphylococcus aureus* (Figure 3A), *Escherichia coli* (Figure 3B), and *Streptococcus mutans* (Figure 3C). Chlorhexidine, widely considered the “gold standard,” was used as a positive control. An MTT assay and UV–VIS spectrophotometry at 595 nm were conducted to evaluate the remaining viable bacteria after 4 h of exposure. No antibacterial effect was found against any of the strains investigated.

### 3.4. Differentiation of DPSCs into Adipogenic, Chondrogenic, and Osteogenic Lineages

**Figure 4:**
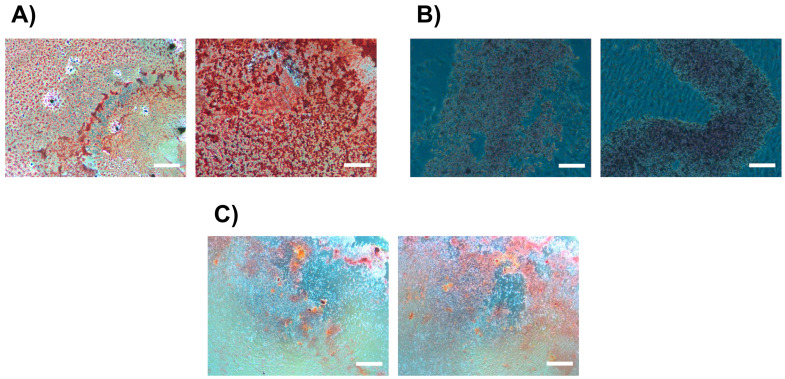
Characterization of stem cells derived from dental pulp stem cells (DPSCs). (**A**–**C**) Photographs taken using phase-contrast microscopy, left row = 0 μM, right row = 8.03 μM of purified caffeine. Adipogenic (**A**), chondrogenic (**B**), and osteogenic (**C**) differentiation of DPSCs after caffeine treatment. Adipocytes were stained with Oil Red O (0.3%) in 60% isopropanol, chondrocytes with Safranin O (0.1%), and osteocytes with Alizarin Red S, confirming lineage-specific differentiation under respective induction conditions. Scale bars = 1416 μm.

To determine whether the dose used was sufficient to promote adipogenic and chondrogenic differentiation—while remaining low enough to avoid negatively affecting osteogenic differentiation—a differentiation assay was conducted following exposure to 8.03 μM of purified caffeine. The results were qualitatively assessed using staining and evaluated under phase-contrast microscopy. Adipogenic differentiation was assessed by detecting reddish-orange lipid deposits using Oil Red O staining (Figure 4A). Chondrogenic differentiation was evaluated by detecting glycosaminoglycans stained blue with Safranin (Figure 4B). Osteogenic differentiation was assessed by detecting reddish mineral deposits stained by Alizarin Red S (Figure 4C). As shown in Figure 4, caffeine produced a significant increase in adipocyte proliferation, with no visible differences in either osteogenic or chondrogenic lineages. 

### 3.5. COX-1 and -2 Expression

**Figure 5:**
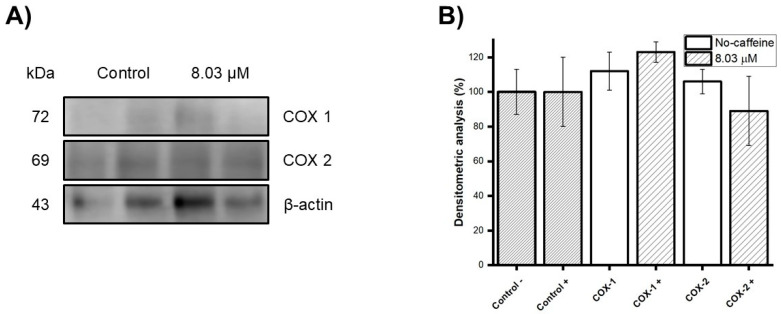
Changes in protein levels in dental pulp stem cells (DPSCs) treated with 8.03 μM purified caffeine. (**A**) Immunoblot detection of β -actin, COX-1, and COX-2 expression in DPSCs. (**B**) Densitometric analysis of the Western blot bands. (Control = β–actin, (+) groups treated with 8.03 μM of purified caffeine). Data are expressed as the percentage of control ± S.D. Statistical significance was determined using one-way ANOVA. No statistically significant differences between groups were observed.

DPSCs were treated with 8.03 μM of purified caffeine after exposure to IL-33 to promote inflammation. They were subjected to electrophoresis, immunoblotting, and immunodetection. The resulting bands are illustrated in Figure 5A, and a densitometric analysis of the bands was performed using Image-J software version 1.54p (LOCI University of Wisconsin, Madison, WI, USA) (Figure 5B). Results show no statistically significant differences between the groups treated with 8.03 μM of purified caffeine vs the untreated groups.

## 4. Discussion

Periodontal disease is a chronic inflammatory condition characterized by the progressive destruction of the tissues that provide support to the teeth. This process results from a complex interplay between dysbiotic microbial communities and an aberrant host immune response [24,25,26,27,28]. 

Caffeine is widely consumed in various forms, including foods, beverages, medications, and cosmetics [29]. It is a white crystalline xanthine that acts as a stimulant of the nervous system. In addition to enhancing alertness, mood, and athletic performance [6], caffeine has also been reported to exhibit anti-aging, antioxidant, and anti-obesity properties [8]. However, it can cause adverse effects such as sleep disturbances, anxiety, and hyperactivity [6]. In the medical field, caffeine is commonly used in combination with analgesics, nonsteroidal anti-inflammatory drugs, and muscle relaxants [30]. In the United States, over 85% of the population consumes caffeine daily, with an average daily intake of 135 mg. In other countries, such as Argentina and Brazil, the estimated daily intake is approximately 100 mg and 40 mg, respectively. The primary sources of caffeine consumption are coffee and tea [6], with caffeine content ranging from 30 to 175 mg per 150 mL of coffee. In Mexico, the average per capita coffee consumption is approximately 2.6 cups per day [31]. Approximately 10% of global coffee demand is for decaffeinated coffee. The decaffeination process generates a by-product known as “crude caffeine,” which, after purification, yields pure caffeine [7].

To our knowledge, no experiments have specifically explored the effects of caffeine on oral cells. The few studies conducted on caffeine in other cell types often present contradictory findings, suggesting that caffeine’s effects are highly dependent on the specific cell type being investigated [12,13,14].

This study aimed to identify a concentration of caffeine capable of delivering its reported biological benefits while minimizing—or, if possible, avoiding altogether—any negative effects. Dental pulp stem cells (DPSCs) were selected due to their accessibility and superior biological characteristics compared to periodontal ligament stem cells (PDLSCs) [21,32,33,34]. DPSCs are easier to isolate in larger quantities from extracted teeth, exhibit higher proliferative capacity, and demonstrate robust osteogenic and odontogenic differentiation potential. In contrast, PDLSCs, while relevant for periodontal regeneration, are more limited in number and more difficult to obtain in sufficient yield. Moreover, DPSCs have shown a broader immunomodulatory profile and higher plasticity, making them a more versatile and practical cell model for in vitro studies assessing the effects of bioactive compounds such as caffeine.

To ensure the accuracy of the experimental outcomes, purified caffeine was used, allowing the observed effects to be directly attributed to the alkaloid itself rather than to confounding components commonly present in caffeine-containing sources.

The first challenge addressed was to avoid the decrease in cell viability previously reported by other authors [8]. A metabolic activity assay was performed to determine the viability thresholds of DPSCs exposed to various concentrations of purified caffeine. Caffeine is known to interfere with cell cycle progression, metabolism, and lysosomal function, as well as induce apoptosis [15,35]. Its cytotoxic potential varies depending on the dose and cell type [13,15,36,37]. All the concentrations tested in this study resulted in cell viability levels above 75%, which can be classified as “Non-cytotoxic” according to the ISO 10993-5 [22]. However, the highest concentration tested (8.03 μM) demonstrated a notable decrease in the cell viability levels compared to the next dose (4.01 μM), with viability dropping from 99% to 86%. This suggests that DPSCs may be more sensitive to caffeine-induced cell viability reduction than other cell types previously studied. To assess the long-term effects, cell proliferation experiments were conducted over a period of up to 21 days. Results showed that 8.03 μM and 4.01 μM of purified caffeine did not negatively affect cell proliferation, as both concentrations maintained growth comparable to that of the control groups. Previous studies have suggested that caffeine may contribute to cell cycle arrest at the G1/S phase by acting on the cyclin D1/Cdk4 complex, depending on the cell type and dose [38].

Caffeine has been shown to possess antibacterial effects against strains such as *Escherichia coli*, *Staphylococcus aureus*, and others, at an MIC between 62.5 and 250 μg/mL [9]. Antibacterial activity against *Streptococcus mutans* has also been reported at concentrations greater than 2 mg/mL [9,10]. However, these studies employed concentrations considerably higher than those employed in this study (1–8.03 μM). The results show that caffeine, at the tested concentrations, exhibited no antibacterial effect against *Streptococcus mutans*, *Escherichia coli*, or *Staphylococcus aureus*. It not only failed to produce results comparable to the gold standard, chlorhexidine, but in some cases even appeared to promote bacterial growth. These findings suggest that the concentrations used in this study were insufficient to induce an antibacterial effect. This observation is consistent with previous reports involving the same bacterial species and similar caffeine concentrations, where no antibacterial activity was detected [39]. Although an antibacterial effect would be desirable, increasing the dose to achieve it would compromise cell viability—a trade-off that is not acceptable within the objectives of this investigation. 

From a regenerative medicine perspective, the ability to promote cell differentiation is arguably the most critical aspect of this investigation. Adipogenic, chondrogenic, and osteogenic differentiation were the bases upon which the potential of purified caffeine for DPSCs characterization was tested. These three lineages represent key mesenchymal pathways relevant for dental tissue regeneration, including the formation of alveolar bone, periodontal ligament, and pulp-dentin complex structures. The successful induction of these phenotypes supports the hypothesis that caffeine, at specific concentrations, may enhance the regenerative performance of DPSCs. This outcome lays the groundwork for future strategies in dental tissue engineering, where caffeine could be used as a bioactive supplement in scaffold-based therapies or incorporated into functional biomaterial systems aimed at restoring dental and periodontal structures through biologically guided regeneration. Controversial effects have been reported regarding the impact of caffeine on the characterization of these lineages. Caffeine has been reported to reduce chondrogenic and osteogenic differentiation by downregulating factors related to them, such as SOX-9 in chondrogenesis [15], and Runx2, OCN, and Osterix in osteogenesis [12]. However, it is important to note that such findings have been reported at much higher doses of caffeine (0.01–2 mM), which were found to reduce cell viability in a significant manner. Conversely, caffeine has been shown to enhance adipogenic differentiation by upregulating related genes such as Pparg, LPL, and Fabp4 [12]. In this study, 8.03 μM promoted adipogenesis and had no observable negative impact on chondrogenesis or osteogenesis. Given the importance of adipogenesis in regenerative medicine, these results highlight the potential utility of this xanthine in the field of tissue engineering. Recent evidence supports the role of adipocytes not only as differentiated endpoints, but also as active contributors to tissue regeneration. As described by García-Urkia et al. [40], adipocytes and adipose-derived components can enhance osteogenic responses, support scaffold remodeling, and play a crucial role in the development of bioengineered teeth and maxillofacial structures. Their involvement in modulating the extracellular matrix and providing trophic support positions adipogenic differentiation as a strategic target in dental tissue engineering. Therefore, the ability of caffeine-primed DPSCs to undergo adipogenesis may represent an added advantage in the design of composite regenerative therapies for craniofacial applications.

Since inflammation is one of the main concerns in dentistry, it was essential to ensure that caffeine would not exacerbate it. Limited research has been conducted on the impact of caffeine on inflammatory processes in vitro. Caffeine has been reported to inhibit the expression of pro-inflammatory cytokines such as COX-2 [8], and other inflammatory-mediating factors such as IL-6, at concentrations of 0.3 to 0.1 mM [41]. According to previous reports, it also suppresses the pro-inflammatory response through p38MAPK phosphorylation and NF-κB activation [8]. In this study, caffeine at a dose of 8.03 μM did not significantly affect the expression of COX-1 and 2 in DPSCs, suggesting a neutral role in inflammatory modulation at this concentration, in this cell type.

These findings suggest promising therapeutic applications in both pulpal and periapical pathologies, where regenerative approaches may enhance healing beyond conventional endodontic treatment [42]. DPSCs, when combined with bioactive scaffolds, could support bone regeneration [43] and modulate chronic inflammation in apical lesions [44]. Additional strategies—such as growth factor delivery, nanoparticle-enhanced antimicrobial systems, and scaffold-based tissue engineering—represent emerging tools for promoting periapical tissue repair [45,46,47]. These innovative approaches shift the focus from infection control alone to biologically driven regeneration of both pulpal and periapical tissues [45].

The future perspective of using caffeine for regenerative purposes must be supported by further evaluations, including LDH assays to directly assess cytotoxicity, as well as additional studies on its long-term effects on cellular differentiation, inflammation modulation, and tissue-specific responses. In relation to adipogenic differentiation, it is acknowledged that the inclusion of molecular markers such as PPARγ2 and CEBPQ would provide a more comprehensive understanding of adipocyte characterization. Although these analyses were not performed in the present study due to technical constraints, their relevance is recognized, and they have been considered as part of future research directions.

## 5. Conclusions

This study determined that purified caffeine, at concentrations up to 8.03 μM, is biocompatible with DPSCs, supporting cell viability and proliferation over a 21-day period. While no antibacterial effects were observed, there were also no adverse inflammatory responses or suppression of chondrogenic or osteogenic differentiation. Most notably, caffeine significantly enhanced adipogenic differentiation, positioning it as a potential agent for dental applications. Further studies are recommended to quantify differentiation markers across multiple lineages and to investigate caffeine’s interactions with a broader range of microbial species.

## Figures and Tables

**Table 1:** Summary of culture media and conditions used for the induction of DPSC differentiation into adipogenic, chondrogenic, and osteogenic lineages.

Lineage	Adipogenic	Chondrogenic	Osteogenic
Composition of the differentiation medium	D-MEM, dexamethasone (0.1 mM), β-glycerophosphate (10 mM), ascorbic acid (50 μg/mL), insulin and L-Glutamine	D-MEM, dexamethasone (0.1 mM), β-glycerophosphate (10 mM), ascorbic acid (50 μg/mL), BMP-4	D-MEM, dexamethasone (0.1 mM), β-glycerophosphate (10 mM), ascorbic acid (50 μg/mL)
Differentiation time	4 weeks	2 weeks	4 weeks
Staining	Oil Red (0.3) + isopropanol 60% *v*/*v* (100 mL)	Safranin (0.1%)	Alizarin Red S
Staining time	1 h	5 min	10 min

## Data Availability

The original contributions presented in the study are included in the article, further inquiries can be directed to the corresponding authors.

## Abbreviations

The following abbreviations are used in this manuscript:

μM	Micromole
*Strep. mutans*	*Streptococcus mutans*
*Esch. coli*	*Escherichia coli*
*Staph. aureus*	*Staphylococcus aureus*
DPSC	Dental pulp stem cells
COX-2	Cyclooxygenase 2
SC	Stem cells
BMMSC	Bone marrow mesenchymal stem cells
CFU-F	Colony-forming unit-fibroblast
ENES Leon	Escuela Nacional de Estudios Superiores, Unidad León
LII	Laboratorio de Investigación Interdisciplinaria
PBS	Phosphate buffer saline
EDTA	Ethylenediaminetetraacetic acid
h	Hours
MTT	3-(4, 5-dimethylthiazol-2-yl)-2, 5-diphenyltetrazolium bromide
mg/mL	Milligrams per milliliter
MEM	Minimal essential medium
min	Minutes
DMSO	Dimethyl sulfoxide
nm	nanometers
ISO	International Organization for Standardization
mL	Milliliters
μL	Microliters
°C	Celsius degrees
D-MEM	Supplemented minimal essential medium
BMP-1	Bone morphogenetic protein 1
IL-33	Interleukin 33
SDS-PAGE	Sodium Dodecyl Sulfate-Polyacrylamide Gel Electrophoresis
PVDF	Polyvinylidene Fluoride
COX-1	Cyclooxygenase 1
HRP	Horseradish peroxidase
UV-VIS	Ultraviolet visible
Cdk4	Cyclin-dependent kinase 4
MIC	Minimal inhibitory concentration
μg/mL	Micrograms per milliliter
SOX-9	SRY-related HMG box gene 9
Runx2	Runt-related transcription factor 2
Osterix	Sp7 transcription factor
Pparg	Perioxisome proliferator-activated receptor gamma
Fabp4	Fatty acid-binding protein 4
IL-6	Interleukin 6
p38MAPK	Mitogen-activated protein kinase 38
NF-κB	Nuclear factor kappa B
UV–VIS	Ultraviolet visible
mg	Milligrams
